# CO_2_ Capture with Mesoporous Silicas Modified with Amines by Double Functionalization: Assessment of Adsorption/Desorption Cycles

**DOI:** 10.3390/ma11060887

**Published:** 2018-05-25

**Authors:** Kléver Santiago Sánchez-Zambrano, Lairana Lima Duarte, Débora Aline Soares Maia, Enrique Vilarrasa-García, Moisés Bastos-Neto, Enrique Rodríguez-Castellón, Diana Cristina Silva de Azevedo

**Affiliations:** 1 Grupo de Pesquisa em Separações por Adsorção, Department of Chemical Engineering, Universidade Federal do Ceará, Campus do Pici, Fortaleza 60455760, CE, Brazil; thiago_1014@outlook.es (K.S.S.-Z.); lairanald@hotmail.com (L.L.D.); deboraunsl@gmail.com (D.A.S.M.); enrique@gpsa.ufc.br (E.V.-G.); mbn@ufc.br (M.B.-N.); 2 Department of Inorganic Chemistry, Crystallography and Mineralogy, Universidad de Málaga, Campus de Teatinos, 29071 Málaga, Spain; castellon@uma.es

**Keywords:** silica, CO_2_ adsorption, double functionalization

## Abstract

CO_2_ adsorption on mesoporous silica modified with amine by double functionalization was studied. Adsorption microcalorimetry was used in order to investigate the influence of increasing the nitrogen surface density on double functionalized materials with respect to the only grafted materials. The distribution of sites and the rate-controlling mechanism of adsorption were evaluated. A Tian Calvet microcalorimeter coupled to a manometric setup was used to evaluate the energy distribution of adsorption sites and to calculate the thermokinetic parameters from the differential enthalpy curves. CO_2_ and N_2_ adsorption equilibrium isotherms at 50 and 75 °C were measured with a magnetic suspension balance, allowing for the computation of working capacity and selectivity at two temperatures. With these data, an Adsorbent Performance Indicator (API) was calculated and contrasted with other studied materials under the same conditions. The high values of API and selectivity confirmed that double functionalized mesoporous silica is a promising adsorbent for the post combustion process. The adsorption microcalorimetric study suggests a change in active sites distribution as the amine density increases. Maximum thermokinetic parameter suggests that physisorption on pores is the rate-controlling binding mechanism for the double-functionalized material.

## 1. Introduction

The emission of greenhouse gases poses a challenge on governments, researchers, and the population around the world because of its possible effects on the planet climate change. As a result, in November 2017, COP23 was held in Germany, when strategies to reach the goals of the global action plan to combat global warming were discussed, aiming at efforts to limit the Earth’s temperature increase to below 2 °C.

Anthropogenically generated CO_2_ is considered to be one of the major greenhouse gases responsible for global warming, primarily due to the combustion of fossil fuels for energy production, which accounts for more than 65% of global CO_2_ emissions [1,2]. At this scenario, large sources of greenhouse gases (GHG) come from burning fossil fuels, like petroleum, mineral coal, and natural gas, all of them arising mainly from the energy, industry, and transportation sectors [3]. Thereby, Carbon Capture, Utilization, and Storage (CCUS) applied to flue gases is expected to be a viable alternative to reduce the emissions of CO_2_, which is a major GHG [4,5,6]. Thus, preventive and remedial methods to deal with those emissions are currently under investigation, among which stand out absorption, cryogenic, and adsorption processes.

Absorption processes utilizing liquid amines show high rates of carbon capture and are widely used in industrial scale, however, there are disadvantages that are associated to their corrosive potential, such as the high amount of energy required for amine regeneration and amine losses during operation [7,8]. Therefore, other technologies for CO_2_ separation from flue gas have been sought. Porous solid adsorbents have been widely investigated as a medium for CO_2_ separation. Among these adsorbents, zeolites 4A, 13X, ZSM-5 [9,10,11], activated carbons [12,13,14], and Metal Organic frameworks (MOF’S) [15,16,17] have been considered for low temperature applications. However, these adsorbents suffer from a rapid decline in adsorption capacities, with increases in temperature despite their high CO_2_ adsorption capacities at room temperature. In addition, their selectivity for CO_2_ in the presence of other gases, such as N_2_, is low. The high energy input that is required to regenerate some microporous adsorbents (e.g., zeolites) is also a serious disadvantage. Therefore, more selective and efficient CO_2_ adsorbents have been widely investigated, as in the case of porous supports that are functionalized with organic molecules that contain amino groups. Grafting and impregnation are commonly used techniques to incorporate organic molecules that contain amino groups on mesoporous silica supports [18,19,20,21,22,23,24,25]. The efficiency of grafting is related to the availability of OH groups on the solid surface and the density of nitrogen in the grafted moiety [18,19,20]. In spite of generally reaching lower incorporated nitrogen concentration than impregnation, the pending amino groups are generally easily accessible by CO_2_. In impregnation, the organic load is much higher; however, because impregnated molecules are stacked inside narrow pores, there may be diffusional limitation. Amino groups may be less accessible, which leads to lower CO_2_/N molar ratios [21]. Sanz et al. [22] reported a double-functionalized material with CO_2_/ N up to 0.48, presenting a high efficiency of the incorporated amino groups for CO_2_ adsorption and claimed its stability in vacuum and temperature, which makes the regeneration process easier.

Several spectroscopic techniques have been used to study CO_2_−amine interactions, with Fourier Transform Infrared Spectroscopy (FT-IR) and Nuclear Magnetic Resonance (NMR) being the most outstanding [23,24,25,26]. Although these spectroscopic experiments are able to identify the nature of the active site, they are not applicable to measuring the energy distribution of sites in adsorption; as such, additional complementary techniques need to be explored.

Previous works have demonstrated that the measurement of adsorption isotherms via method manometric device in a customized Tian-Calvet calorimeter can be used successfully to measure the heats that evolved upon CO_2_ adsorption [27,28]. It has been found that the textural characteristics of the support and the nature/ density of the functionalized moiety have significant effects on the heat of adsorption as a function of coverage. Using calorimetry, it has been shown that there are multiple amines to interact with one CO_2_ molecule under dry conditions, forming strong alkylammonium carbamate species (~90 kJ mol^−1^) [29] when the amine density is sufficiently high (>1.5 mmol Ng^−1^).

In this work, the changes in site energy distribution and kinetic mechanism have been assessed by adsorption microcalorimetry for mesoporous silicas that were previously grafted with (3-aminopropyl) triethoxysilane (APTES), and then impregnated with polyethyleneimine (PEI). The double functionalized and the simply grafted sample were also tested using a magnetic suspension balance at temperatures close to post combustion scenario in order to investigate their adsorption capacity at these conditions. At the end, the adsorbent with a high Adsorbent Performance Indicator was studied in three cycles of regeneration, in order to contrast the energy consumption that is required to reach complete desorption and the new adsorption capacity in isothermal condition after each adsorption/desorption cycle was measured.

## 2. Materials and Methods

### 2.1. Materials

#### 2.1.1. Gases

The gases used as adsorbates in adsorption measurements and microcalorimetric studies were helium (White Martins Praxair, Inc., São Paulo, Brazil, 99.999%), carbon dioxide (White Martins Praxair, 99.8%), and nitrogen (White Martins Praxair, 99.999%). Nitrogen was also used to determine the textural properties from N_2_ adsorption/desorption isotherms at −196 °C. Helium was used for calibration procedures. 

#### 2.1.2. Synthesis of Mesoporous Silica

The synthesis of pure mesoporous silica (MSS) was performed using a hydrothermal route, as described by Fulvio et al. [30], with some minor modifications. Briefly, 5.7 g P123 (Sigma Aldrich, São Paulo, Brazil) was used as a structure-directing agent and 0.065 g NH_4_F (Sigma Aldrich, São Paulo, Brazil) as a swelling agent to reduce the length of the channels [31]. They were mixed in 200 mL HCl solution (1.3 mol L^−1^) (Labsynth, Diadema, SP, Brazil) and were stirred at room temperature until the complete dissolution of the surfactant. Then, 12.2 g TEOS (Sigma Aldrich, São Paulo, Brazil) was added as silica source and it remained under stirring for 24 h at room temperature. The solution was then transferred to a Teflon lined reactor and heated at 100 °C for 48 h.

After that, the solids were filtered, washed, and dried at 100 °C for 24 h. The dried solids were then calcined at 550 °C at a heating rate of 2 °C min^−1^ for 5 h.

#### 2.1.3. Grafting with APTES

(3-aminopropyl)triethoxysilane (APTES) grafting on pure mesoporous silica (MSS) was carried out following the methodology described by Hiyoshi et al. [32]. The pure mesoporous silica (2.0 g), previously dried at 110 °C, was introduced into a three-neck flask with 20% APTES (Sigma Aldrich, São Paulo, Brazil) solution (*v/v*) in toluene (Labsynth, Diadema, SP, Brazil). The solution was heated overnight under reflux at 110 °C in inert atmosphere (i.e., N_2_ atmosphere). Then, the grafted silica was filtered and washed with toluene three times and then finally dried at 100 °C. The obtained sample was named as MSG20. The number in this label represents the volume percentage of APTES in toluene in the grafting step.

#### 2.1.4. Impregnation with Polyethylenimine (PEI)

In this step, the grafting and impregnation methods were combined to obtain a higher nitrogen load (as compared to MSG material) and a higher mobility of some amino groups [22].

MSG20 was the starting material. Following the wet impregnation method that was used by Xu et al., 2002 [33], 0.40 g PEI 50% *m*/*v* in water (Fluka Analytical, Buchs, Switzerland) were stirred with 3.6 g of methanol (Labsynth, Diadema, SP, Brazil) for about 15 min. Then, 0.45 g of MSG20 were added to the solution, maintaining a proportion of 8 g of methanol per gram of MSG20 sample [34]. The resulting slurry was continuously stirred for about 30 min and the solid was dried at room temperature overnight. The as-prepared adsorbent was denoted as MSG20I30, where 30 represent the loading of PEI as the weight percentage of the sample.

### 2.2. Chemical and Textural Characterization

The chemical composition in terms of C, H, and N content in the samples was obtained by elemental analysis and it was performed using a CHNS/O Analyzer 2400, Series II, from Perkin Elmer (Norwalk, CT, USA). The density of amino groups, $\emptyset_{-{\mathrm{NH}}_{2}}$ [Amine molecules nm^−2^] was calculated by means of the nitrogen concentration using Equation (1). This equation is a modification of that previously reported by Liu et al. [35].

(1) $$\emptyset_{-{\mathrm{NH}}_{2}}=\frac{\mathrm{Nc}\cdot N_{A}}{S_{\mathrm{BET}}\cdot 10^{18}}$$

where $\emptyset_{-{\mathrm{NH}}_{2}}$ is the amine density (molecules nm^−2^), N_A_ is the Avogrado number, N_c_ is the nitrogen content (mol g^−1^) that was obtained from elemental analysis and S_BET_ is the specific surface area (m^2^ g^−1^).

Textural properties of silica samples were estimated from N_2_ adsorption/desorption isotherms at −196 °C using an Autosorb iQ3 (Quantachrome Instruments, Boynton Beach, FL, USA). MSG20 and MSG20I30 were outgassed at 120 °C under vacuum (10^−6^ bar) during 4 h. Specific surface area of all the materials was calculated using Brunauer-Emmett-Teller (BET) equation [36] and micropore volume by Dubinin–Radushkevich (DR) equation [37]. Pore size distribution (PSD) of each sample was obtained using the BJH method [38] while using the desorption branch. The total pore volume was calculated from the adsorption isotherm at P/P_0_ = 0.985.

X-ray powder diffraction patterns (XRD) were collected on X-ray diffractometer model X’Pert Pro MPD (PANalytical, Almelo, The Netherlands), with a Ge (1 1 1) primary monochromator (strictly monochromatic Cu-Kα radiation source, λ = 1.5406 Å) with a X’Celerator (Real Time Multiple Strip) detector that was equipped with 128 Si aligned detectors. 

Transmission electron micrographs (TEM) were obtained by using a Philips CM 200 Supertwin-DX4 microscope (FEI, Hillsboro, OR, USA). Samples were dispersed in ethanol and a drop of the suspension was placed on a 300-mesh Cu grid.

Thermogravimetric analyses (TGA) were carried out using as equipment model STA 409 CD/403/5/G SKIMMER (Netzsch, Selb, Germany) with a heating rate of 10 °C min^−1^, under synthetic air flux (20 mL min^−1^), with approximately 5.0 mg of sample. The temperature range for the TG analyses was from room temperature up to 800 °C.

#### 2.2.1. Microcalorimetric Experiments

The samples were previously outgassed (10^−3^ mbar) at 120 °C for 4 h. A Setaram C80 microcalorimeter (Setaram, Caluire, France) that was internally composed of an array of thermocouples was used. This setup is coupled to a manometric adsorption system. This system is used to measure the quantity of gas adsorbed in equilibrium under isothermal conditions and obtain the differential adsorption enthalpy. For each gas injection, the adsorption enthalpy was calculated using the so-called discontinuous procedure, as described by Rouquerol et al. [39]. The integration of heat peaks was realized by Calisto^®^ Software (v1.043 AKTS-Setaram, Caluire, France).

This heat peak may provide not only thermodynamic, but also kinetic information, as mentioned by Stošić and Auroux (2013) [40]. The kinetics of heat release during adsorption can be monitored by the change in the thermokinetic parameter *τ*. The calorimetric signal (*D*) decreases exponentially with time (*t*) after the maximum of each adsorption peak. Equation (2) shows the linearized form of this exponential decay, from which the thermokinetic parameter *τ* may be estimated.

(2) $$\log\left(\frac{D}{D_{m}}\right)=-\frac{t}{\tau }$$

where *D* and *D_m_* represent the power signal and the maximum power signal (mW), *t* is the time, and *τ* is the thermokinetic parameter, both in seconds.

According to the model that was described by Cardona and Dumasic (1992) [41], the curve of adsorption enthalpy as a function of uptake can be fitted by a polynomial function (Equation (3)). The reciprocal of the first derivative (Equation (4)) would lead to the distribution of energetic sites as a function of the adsorption enthalpy. Therefore, the energy distribution function *f*(*q*) is plotted for sites with similar energy, where *n* is the number of moles adsorbed in each of these sites and *a_i_* are the polynomials coefficients. If we integrate this distribution in a given range of enthalpies, then we can obtain the density of energy sites that are available to adsorption in this enthalpy range.

(3) $$\Delta h_{diff}=\frac{\Delta h_{int}}{dn}=\sum_{i=0}^{k}a_{i}n^{i}$$

(4) $$f\left(q\right)=-\frac{dn}{d\Delta h_{diff}}=-\frac{1}{\sum_{i=1}^{k}ia_{i}n^{i-1}}$$

#### 2.2.2. Adsorption Equilibrium

A magnetic suspension balance (Rubotherm, Bochum, Germany) was used to obtain equilibrium experimental data of pure CO_2_ and N_2_ at 50 and 75 °C and a pressure range of 0.01–10 bar. Binary gas isotherms were also obtained with a composition of 15% CO_2_/85% N_2_ *v*/*v* (close to post combustion scenario). Prior to the measurements, the samples were outgassed under vacuum (0.01 bar) at 120 °C for 4 h.

The excess amount of adsorbed gas was calculated by using Equation (5). The microbalance senses the resulting force that is acting on the sample ∆*m* (*P*,*T*).

(5) $$m_{ex}\left(P,T\right)=\Delta m\;\left(P,T\right)+\left(V_{B}+V_{S}\right)\cdot \rho_{g}\left(P,T\right)$$

where *m_ex_*(*P*,*T*) is the excess adsorbed mass and ρ*_g_*(*P*,*T*) is the gas density evaluated by means of an equation of state. *V_B_* and *V_s_* (cm^3^) are the volume of the suspended parts in the measuring cell and solid volume, respectively, with both being determined with Helium essays. 

Dual site Langmuir model (Equation (6)) was used to fit the experimental CO_2_ adsorption data. It considers that the gas is adsorbed in two different sites, one where chemisorption has an important contribution (site 1) and the other one where physisorption is the predominant mechanism (site 2). For N_2_ adsorption experimental data, a simple Langmuir model was used.

(6) $$q=\frac{q_{m1}\cdot b_{1}p_{1}}{1+b_{1}p_{1}}+\frac{q_{m2}\cdot b_{2}p_{2}}{1+b_{2}p_{2}}$$

The Multi-Region Extended Langmuir was the model used for multicomponent adsorption equilibrium. This model also considers the existence of two different sites: site 1 that only adsorbs the component with more affinity (CO_2_) while considering chemisorption as dominant mechanism (Equations (7) and (8), respectively) and site 2 that adsorbs both adsorbates (CO_2_ and N_2_), assuming that there is a competition between them, as shown in Equations (9) and (10).

(7) $$q_{CO_{2},1}=\frac{q_{m1CO_{2}}\cdot b_{1CO_{2}}p_{CO_{2}}}{1+b_{1CO_{2}}p_{CO_{2}}}$$

(8) $$q_{N_{2},1}=0$$

(9) $$q_{CO_{2},2}=\frac{q_{m2CO_{2}}\cdot b_{2CO_{2}}p_{CO_{2}}}{1+b_{2CO_{2}}p_{CO_{2}}+b_{2N_{2}}p_{N_{2}}}$$

(10) $$q_{N_{2},2}=\frac{q_{mN_{2}}\cdot b_{N_{2}}p_{N_{2}}}{1+b_{2CO_{2}}p_{CO_{2}}+b_{N_{2}}p_{N_{2}}}$$

The total amount of gas adsorbed *q_T_* (g g^−1^), which is the actual measured variable, is obtained using Equation (11), where $q_{CO_{2},i}$ is the CO_2_ amount that is adsorbed on site 1 and site 2 and $q_{N_{2},2}$ is the nitrogen amount adsorbed on site 2.

(11) $$q_{T}=q_{CO_{2},1}+q_{CO_{2},2}+q_{N_{2},2}$$

The CO_2_/N_2_selectivity was calculated using Equation (12)

(12) $$\alpha_{CO_{2}/N_{2}}=\frac{q_{CO_{2}}}{q_{N_{2}}}\frac{y_{N_{2}}}{y_{CO_{2}}}$$

where $q_{CO_{2}}$ and $q_{N_{2}}$ are the CO_2_ and N_2_ capacity uptakes, respectively. $y_{CO_{2}}$ and $y_{N_{2}}$ are the molar compositions in the gas mixture.

An Adsorbent Performance Indicator (API), as developed by Wiersum et al. [42] (Equation (13)), was calculated for our samples in order to compare them with other samples that were previously reported for CO_2_ post combustion capture. With this objective, working capacity (*WC*) since 0.02 up to 1 bar was calculated, as well as selectivity ($\alpha_{CO_{2}/N_{2}}$) at 50 and 75 °C from mixture isotherms using multi region extended Langmuir model.

(13) $$\mathrm{API}=\frac{{\left(\alpha_{CO_{2}/N_{2}}-1\right)}^{A}WC_{CO_{2}}^{B}}{{\left|\Delta H_{ads,CO_{2}}\right|}^{C}}$$

where *A*, *B,* and *C* are empirical parameters, which may be chosen according to the desired separation/purification process. *WC* is the working capacity of CO_2_; and, Δ*H*_ads_ is the CO_2_ adsorption enthalpy.

## 3. Results

The results of the elemental analysis are summarized in Table 1. These data indicate that nitrogen has been effectively incorporated to the pure MSS sample after functionalization step. 

In addition to nitrogen content, another important result that confirms the presence of amino groups on the grafted and double functionalized samples is the increase of carbon amount. This increase is related to the incorporation of propyl groups of the APTES molecules or/and the alkyl chains of PEI.

Some amount of nitrogen is observed on the MSS sample; a possible explanation might be residual NH_4_F that remained on the material from the synthesis procedure. There is a difference between the C/N ratio measured (~3.3) and expected (3.0) for the MSG20 sample, which is possibly due to the adsorbed atmospheric CO_2_ that increases the carbon amount that is detected by the equipment, as confirmed by 13C-CP-MAS NMR experiments in previous works [26,43].

The Maximum Theoretical Adsorption Capacity (MTAC) by chemisorption is also summarized in Table 1. The highest theoretical chemical adsorption was for MSG20I30 sample. This fact would probably improve the attractiveness of the solid for CO_2_ adsorption.

**Figure 1:**
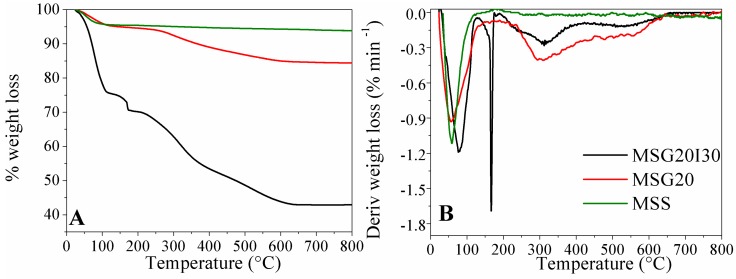
(**A**) Thermogravimetric Analysis (TGA) for mesoporous silica (MSS), MSG20, and MSG20I30 samples; (**B**) derivate of the weight loss (DTGA) for the these materials.

The results of the Thermogravimetric Analysis (TGA) are shown in the Figure 1A. Figure 1B presents the derivate of the weight loss (DTGA) for MSS, MSG20, and MSG20I30 samples.

**Figure 2:**
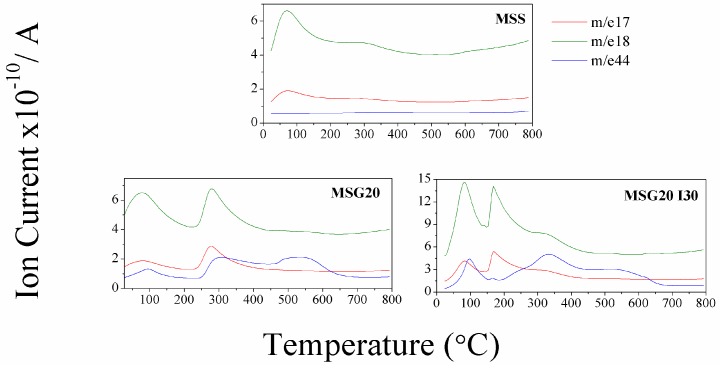
Mass charge ratio distribution measured by TGA.

For all samples, the initial weight loss at around 100 °C is mainly due to the loss of physisorbed water, corresponding to 5% for MSS, 10%, for MSG20 sample, and 25% for MSG20I30 samples (point 1 in Figure 1). The mass spectra analysis, shown in Figure 2, present the signals with *m*/*z* ratio of 17 and 18, which confirms the release of moisture. The difference between the weight loss in the samples can be explained with the additional *m*/*z* 44 that is found in the functionalized samples (MSG20 and MSG20I30), whcih is attributed to the release of the atmospheric CO_2_ adsorbed in the material due to the presence of amine groups. This result is in agreement with the increase in %C observed in elemental analysis.

At 650 °C, the grafted amine was completely decomposed and was removed as volatiles. The organic content (loss weight from 150 up to 750 °C) of MSG20I30 was calculated to be about ~31 wt %, according to PEI load that was employed in the synthesis step. MSG20 has an organic content of around 10%, and this fact indicates that not all PEI dissolved was incorporated during the impregnation step. The maximum operating temperature for MSG20I30 sample would be ~150 °C, in order to avoid the decomposition of the material. This temperature is lower than that of MSG20 sample, in which case, the maximum temperature of operation is ~250 °C.

**Figure 3:**
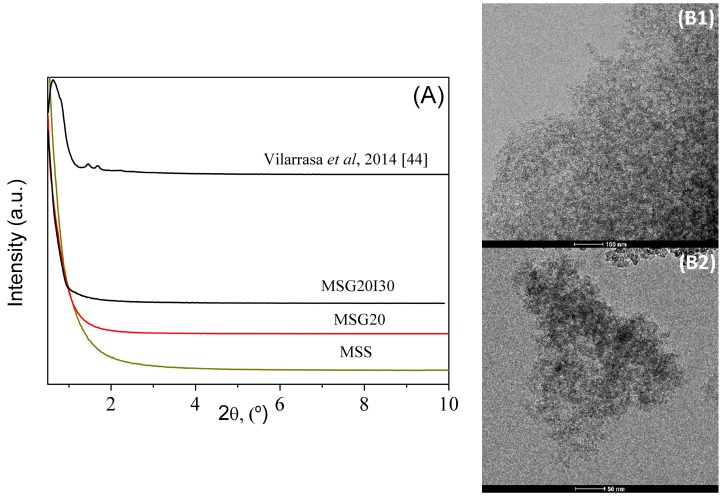
(**A**) X-ray powder diffraction (XRD) patterns of all mesoporous silica samples and transmission electron micrographs (TEM) micrographs of MSS with (**B1**) scale bar = 100 nm and (**B2**) 50 nm.

Low-angle X-ray powder patterns of mesoporous silica MSS, MSG20, and MSG20I30 samples are shown in Figure 3A. The compiled diffractograms are contrasted with a conventional SBA-15 that was previously reported in the literature [44].

Conventional hydrothermal SBA-15 shows a typical XRD pattern of an ordered network of mesopores with (100), (110), and (200) reflections, which are typical of a hexagonal symmetry [44,45]. The characteristic reflections of SBA-15 are not present in our samples. Vilarrasa et al. (2014) [46] and Liu et al. (2012) [47] showed similar behavior as a characteristic of Mesocellular Foam Structure (MSF). The presence of ammonium fluoride on the synthesis process might have affected the hexagonal arrangement of the solid, thus limiting the growth of the mesochannels and leading to shorter channels with low-range order. Ammonium fluoride was employed in the synthesis as a pore swelling agent in order to provide more space for surface functionalization. Many authors [48,49] attribute the conversion of ordered arrangement to mesocellular structure to this type of “precursors”, in which an increase in the pore size is caused by NH_4_F penetration into the hydrophobic core of the surfactant micelle, thus breaking up the typical honeycomb packing of the hydrothermal SBA-15. This fact could be the reason why no noticeable diffraction signals are observed at low-angle. 

Transmission electron micrographs (Figure 3(B1,B2)) show that effectively the addition of NH_4_F prevents the typical hexagonal arrangement of SBA-15, leading to a mesocellular foam structure [46].

**Figure 4:**
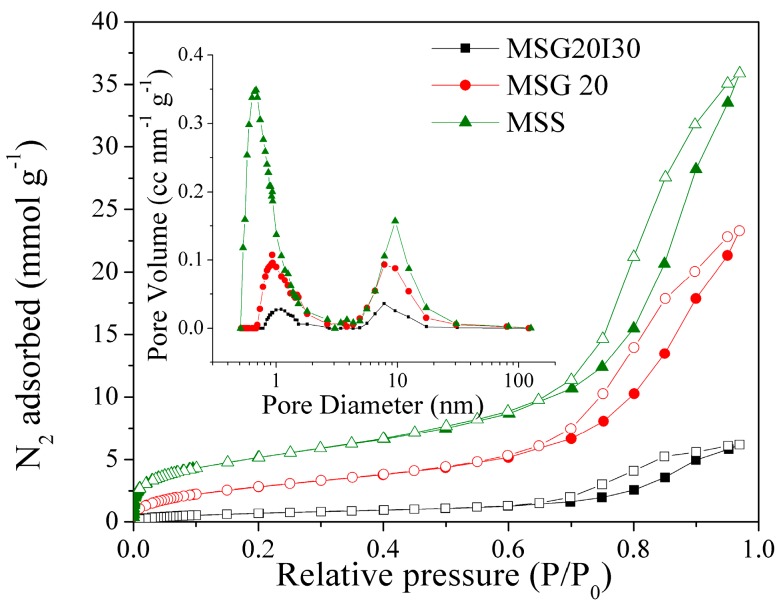
N_2_ adsorption/desorption isotherms and pore size distributions (PSD’s) at −196 °C, open symbols belong to desorption step.

The N_2_ adsorption/desorption isotherms at −196 °C are shown in Figure 4. All of the samples have a H2(b) type hysteresis [50], which is associated with mesocellular silica foams (MSF) [49], leading to a shift of the hysteresis loop to a higher relative pressure. After the immobilization of PEI on MSG20, the total pore volume was reduced from 0.96 to 0.06 cm^3^ g^−1^ (see Table 2). The specific surface area was also reduced dramatically from 211 to 52 m^2^g^−1^, which is expected due to the filling of pores with PEI, decreasing the surface area, micropore, and the total pore volume. The Pore Size Distributions (PSD) for all of the samples in logarithmic scale (inset of Figure 4) show a bimodal distribution with a smaller pore size of ~1 nm (micropores) and larger pores with sizes of around 7.8 nm, confirming that our materials remain mesoporous after the functionalization step.

Textural properties are summarized in Table 2. Textural properties decrease as N content increases, which is indicative that amine groups has been effectively incorporated to the bulk MSS sample.

**Figure 5:**
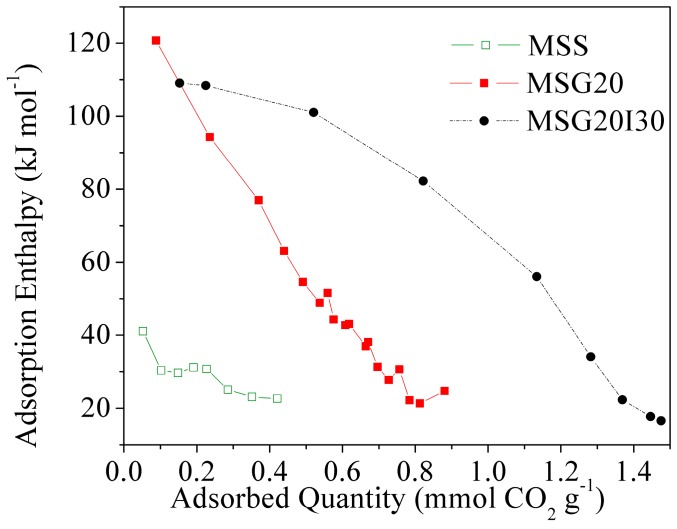
Differential enthalpy of adsorption in function of CO_2_ uptake for mesoporous silica.

Figure 5 shows CO_2_ adsorption microcalorimetric curves of samples at 25 °C under anhydrous conditions. The samples show a decrease in the differential enthalpy with an increasing CO_2_ uptake, which suggests that they have a heterogeneous surface, according to the classification that was proposed by Rouquerol et al. [39].

These curves show that, for the two functionalized samples, the initial enthalpy values are in the range of ~110–120 kJ mol^−1^. Thus, we can consider that this relatively high enthalpy value is due to the interaction of CO_2_ with grafted and/or impregnated amines. Namely, the chemisorption of CO_2_ on amine pairs to form propyl ammonium carbamate species has an adsorption enthalpy of ~−90 kJ mol^−1^ [51].

Although the enthalpy at a low coverage is similar, there is a remarkable change of sites energy with the addition of PEI.

**Figure 6:**
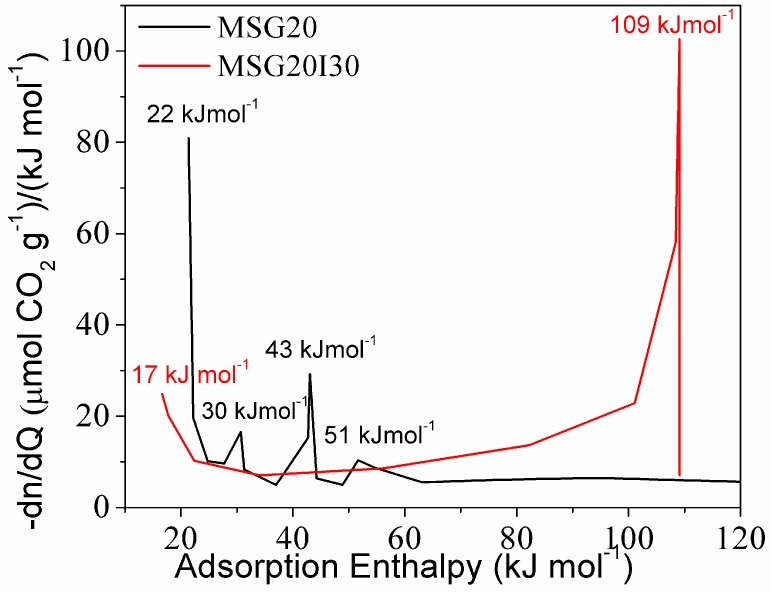
Energy sites distribution.

For a better appreciation of the change of adsorption mechanism on the double functionalized solid, the distribution of active sites adsorbing CO_2_ on the samples is shown in Figure 6. Peaks in the distribution represent the frequency of sites with the same energy of adsorption. In the case of chemisorption, these represent intermediate products that are formed as soon as the CO_2_ pressure increases.

The energy distribution (Figure 6) showed four signals for the MSG20 sample. Two corresponding to low enthalpies are probably related to physisorption. The other two are present at enthalpies that are higher or close to −40kJ mol^−1^ (possibly related to chemisorption). Yoo et al. (2015) [52] mentioned that the enthalpy value of −65kJ mol^−1^ could be associated to the combination of CO_2_ adsorbed via intramolecular interactions with silanols and/or other amine groups (when the grafted moieties are DI-TRI amines) to form carbamate. Therefore, the peak that was observed at the MSG20 energy distribution curve in the range −50–65 kJ mol^−1^ may be attributed to the formation of silyl propyl carbamate on the MSG20 surface.

Moreover, formation enthalpy related to carbamic acid formation (~43 kJ mol^−1^) is also present in the distribution. Bacsik et al. (2011) [53] concluded that the ammonium carbamate ion pairs and hydrogen-bonded carbamic acid were weakly chemisorbed and could be outgassed by vacuum. Danon et al. (2011) [25] observed that, after cell evacuation in FT-IR equipment, only the band associated with the bound (silyl propyl) carbamate kept intact, indicating that this molecule has a stronger interaction that cannot be reversed only by vacuum. For this reason, the MSG20I30 sample could be an interesting CO_2_ capture adsorbent with respect to energy consumption because the irreversibly bound (silyl propyl) carbamate formation is suppressed. MSG20I30 site distribution does not show this peak. On the other hand, diffusional resistances may be increased, as mentioned by Bollini et al. (2012) [21], for materials with high amine density, which affects adsorption kinetics.

The amine density on these samples, as well as the amount of adsorption sites and the maximum thermokinetic parameter (τ_max_) in each case are summarized in Table 3. For both functionalized solids, the active sites with strength lower than −40 kJ mol^−1^ do not vary significantly as the amine density increases, in contrast with the chemisorption sites (strength higher than −40 kJ mol^−1^), which consistently increase for higher amine loadings.

The MSG20 sample showed τ_max_ at −43 kJ mol^−1^, providing additional evidence that the rate-limiting adsorption mechanism is essentially due to carbamic acid formation by hydrogen bonds [18].

For the MSG20I30 sample, the maximum thermokinetic parameter at −34 kJ mol^−1^ is related to physisorption. This provides additional evidence that the adsorption rate-limiting mechanism is essentially due to physisorption on this sample (diffusional resistances).

### 3.1. Pure CO_2_ Adsorption Isotherms at Low Pressures

**Figure 7:**
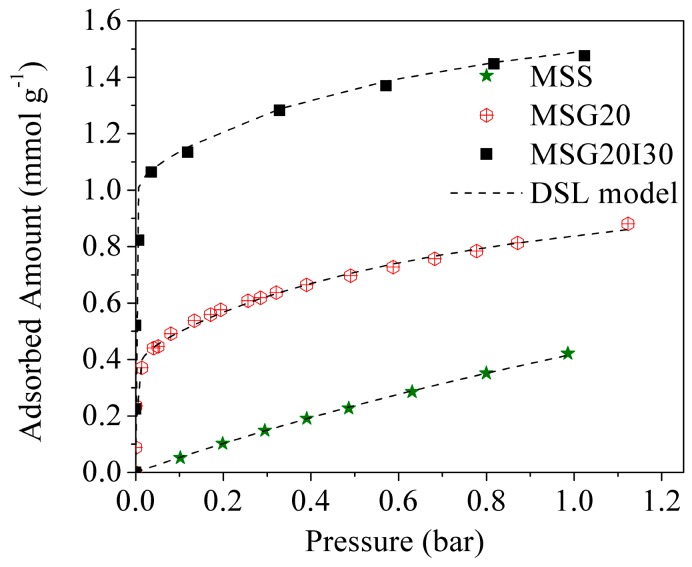
CO_2_ isotherms at 25 °C for mesoporous materials.

CO_2_ adsorption isotherms of all the materials at 25 °C are compared in Figure 7. For the functionalized samples, the isotherms showed a steep increase at pressure <0.1 bar and a gradual increase from 0.1 to 1.0 bar. The high capacity and the steep nature of the CO_2_ isotherm at low pressure on amine loaded silica are known as being caused by the chemical reaction between CO_2_ and the primary amine groups (–NH_2_), forming the products of adsorption previously discussed. The further gradual increase beyond the “knee” from 0.1 to 1.0 bar was attributed to the physical adsorption of CO_2_ on the grafted mesoporous material, but it is more notorious for MSS, which does not present the primary increase knee. As expected, the CO_2_ adsorption at low pressures is more favorable for the double functionalized sample, which has a higher percentage of added amines.

### 3.2. Pure CO_2_ and Binary CO_2_/N_2_ Adsorption Isotherms at High Pressures

**Figure 8:**
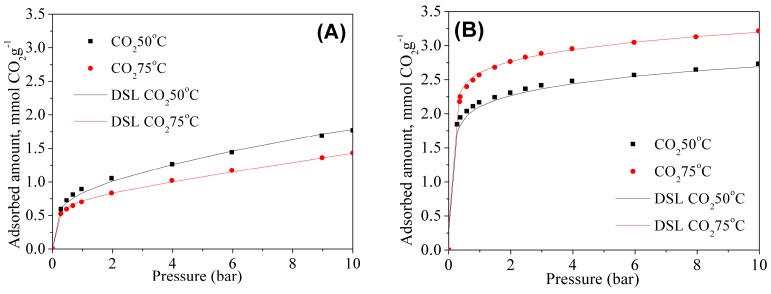
CO_2_ isotherms for (**A**) MSG20 and (**B**) MSG20I30 samples.

High-pressure adsorption isotherms were also measured for grafted and double functionalized material for CO_2_ and N_2_ at 50 and 75 °C. The CO_2_ isotherms are shown in Figure 8A,B. The fitting parameters of Dualsite Langmuir model and the coefficients of determination of the fitted model are summarized in the Table 4 and Table 5 for the MSG20 and MSG20I30 samples, respectively.

As expected, CO_2_ adsorption is enhanced with the increases of the temperature for high density amine sample (MSG20I30), particularly at low pressures. As it can be observed in Table 5, *q_m1_* and *b_1_* are higher at 75 °C for MSG20I30 sample. This behavior is characteristic for chemisorption on samples with high percentages of functionalized amine. The coefficients of determination show for all cases that the model adequately fits experimental data, and this fact has an important impact for the selectivity estimation. 

The results of adsorption capacities that is reported in the literature for mesoporous silica functionalized with APTES, MAP (3-(Methylamino)-propyltrimethoxysilane), and double functionalized with APTES/TEPA and APTES/PEI are summarized in Table 6. The adsorption capacities that were obtained in this work are in the same range as those that are found in the literature under similar conditions.

The MSG20 and MSG20I30 molar selectivities were estimated from binary isotherms using Dual Site Extended Langmuir (DSEL) in order to obtain the CO_2_ adsorbed under binary conditions. The highest selectivity values are reached at low pressures. This is due to the strong interaction of CO_2_ with the incorporated amine, which are mostly available at low pressures. N_2_ at low pressures does not have strong interactions with either –OH or –NH_2_ groups and the physisorption is weak. The highest values for selectivity, as expected, were obtained for MSG20I30 at both temperatures, stressing the advantage of this sample in contrast with the grafted one.

**Figure 9:**
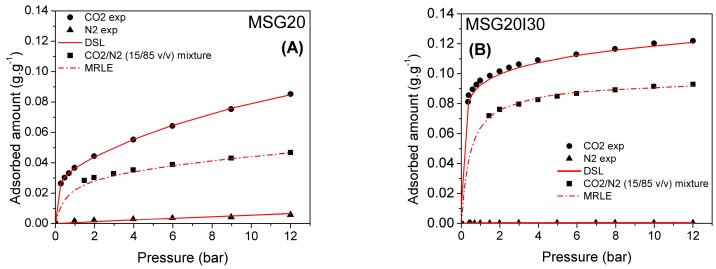
Pure CO_2_ and N_2_ and binary isotherms (0.15 CO_2_ and 0.85 N_2_) at 50 °C for (**A**) MSG20 and (**B**) MSG20I30 samples, continuous lines are fits to Dualsite Langmuir model, and dashed lines are fits to the Multi Region Langmuir Extended model.

Adsorption isotherms for binary mixtures of CO_2_ and N_2_ (15/85 *v*/*v*) are shown in Figure 9 for MSG20 and MSG20I30 samples. The binary mixture mole fraction was chosen to be representative of a post-combustion scenario of flue gases (15% CO_2_/85% N_2_) and at high temperatures (50–75 °C). The points stand for experimental data and the lines stand for predictions from the multi region extended Langmuir (MREL) model using parameters that were obtained from the single component isotherms.

The Adsorbent Performance Indicator (API) was calculated for MSG20 and MSG20I30 samples (see Table 7). In this computation, working capacity in the pressure range from 0.02 bar to 1 bar at 50 and 75 °C was used. The exponents A, B, and C (Equation (13)) were assumed to be 1, following the procedure that was adopted to calculate the API for purification scenarios by Wiersum et al. [42]. The highest value of API is found for the MSG20I30 sample at 75 °C. When the temperature increases, the parameter also does. This indicates a good performance to purification on the post combustion process. The API for MSG20I30 is the highest in contrast with other materials that were studied for the post-combustion scenario. Alvarez-Gutierrez et al. (2017) [59] calculated API for carbons on the post-combustion condition. They obtained values <1 at 50 °C, moreover API for carbons decreases as the temperature increases [60] so that carbons would not be adequate materials for post combustion scenario. Pillai et al. (2015) [61] calculated API on MOFs and they obtained values that were close to the MSG20I30 sample.

### 3.3. Stability and Energy Consumption between Adsorption Cycles

**Figure 10:**
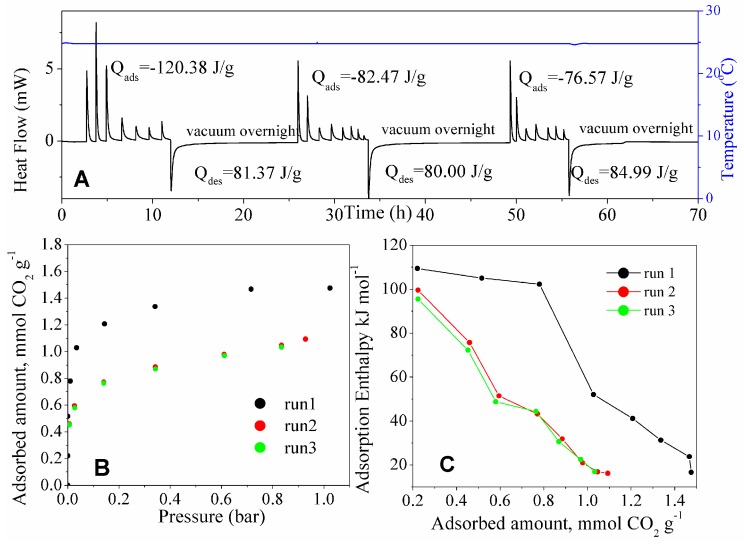
(**A**) Thermogram (at 25 °C) for CO_2_ adsorption on the three rounds; (**B**) corresponding CO_2_ adsorption isotherms; and, (**C**) Differential enthalpies of CO_2_ adsorption (at 25 °C) for the three rounds of adsorption on the same MSG20I30 sample.

**Figure 11:**
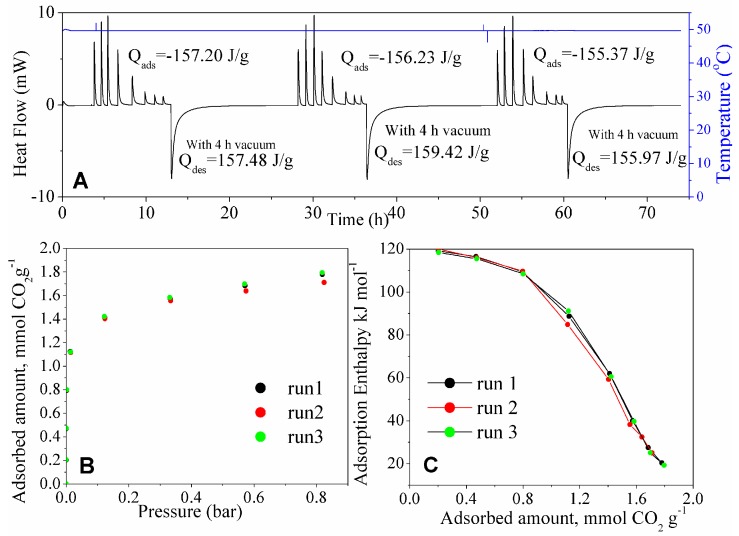
(**A**) Thermogram (at 50 °C) for CO_2_ adsorption on the three rounds, (**B**) corresponding CO_2_ adsorption isotherms, and **(C)** differential enthalpies of CO_2_ adsorption (at 50 °C) for the three rounds of adsorption on the same MSG20I30 sample.

For practical use, the adsorbent should not only possess a high adsorption capacity for pure CO_2_ and a high Adsorbent Performance Indicator, but it should also display a reversible adsorption–desorption pattern. Runs of CO_2_ adsorption (isotherms, thermograms, and differential enthalpies) at 25 and 50 °C on the adsorbent with best performance (MSG20I30 sample) previously degassed at 120 °C for 4 h are shown in Figure 10 and Figure 11, respectively. The CO_2_ adsorption isotherm for run 1 in contrast to run 2 at 25 °C do not follow the same path. The thermograms show a difference between adsorption and desorption enthalpies of 39.01 J per gram of solid. This observation suggests that the CO_2_ molecules that were adsorbed in the first round cannot be completely desorbed, even under overnight molecular vacuum. The enthalpies of adsorption at near-zero coverage do not differ distinctly for the first adsorption and the subsequent ones. This suggests that chemisorption is still happening on the free amine groups that remain after the first evacuation, in lower intensity for adsorption sites occupation. The first occupation of available sites may be due to the irreversible reaction between CO_2_ and amine on this material [62]. On the other hand, that irreversibility may also be attributed to the diffusion limitations that are imposed by the high amine density of MSG20I30 [22,29]. This would result that after the first adsorption run not all CO_2_ is released from the sample during the time high vacuum is applied. It is likely that both mechanisms (chemisorption and hindered diffusion) contribute to cause this irreversibility. 

The calorimetric cycles at 50 °C for MSG20I30 are shown in Figure 11. The thermogram integration shows reversibility at this temperature. The three adsorption isotherms and thermograms overlap at this temperature. An increase of temperature eventually would enhance intraparticle mass transfer, allowing for faster CO_2_ evacuation and have the adsorption sites available again. The rupture of the strong bond formed between CO_2_ and amino propyl groups can be achieved with high temperature and molecular vacuum, as these results at 50 °C suggest. At this temperature, MSG20I30 sample achieves reversibility after 4 h under vacuum. 

**Figure 12:**
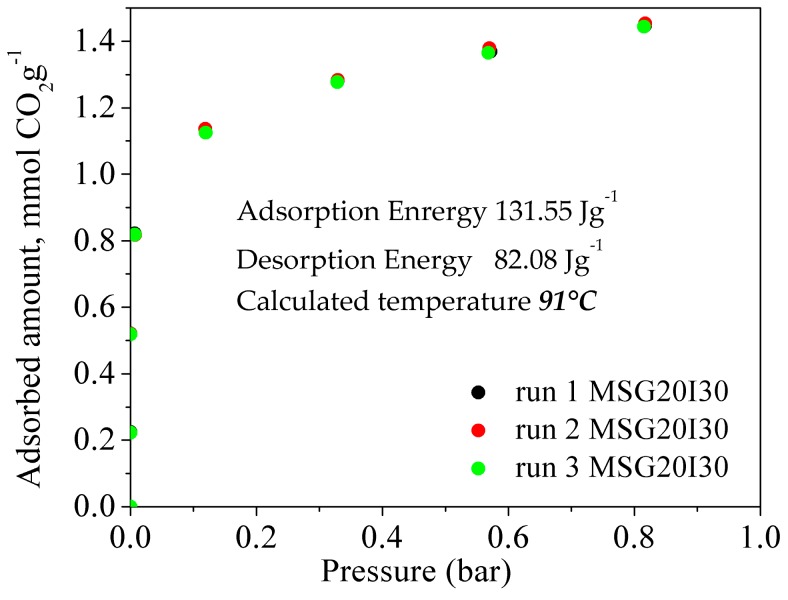
CO_2_ Adsorption Isotherms with regeneration temperature in addition to molecular vacuum, for MSG20I30 sample at 91 °C.

Based on the difference between the energies of adsorption and desorption (after vacuum application), the temperature that is necessary to get complete outgassing was calculated for the experiments carried out at 25 °C, while considering a caloric capacity of 0.75 J g^−1^ °C^−1^. This temperature is in agreement with other works in our group [63], where grafting materials were studied in a fixed bed calculating degassing temperatures around 90 °C with partial pressure reduction. At 50 °C the process is reversible, so this calculation was not computed. Enthalpy data and calculated temperature to complete outgassing are pointed out along with the isotherms that were collected for the three runs for MSG20I30 in Figure 12. 

Three runs at 25 °C for MSG20I30 sample are presented in Figure 12. Both heating up to the calculated temperature and molecular vacuum were used between the runs, with the purpose of testing if adsorption-desorption is truly reversible at these conditions. The results show that the MSG20I30 has reversibility in the pressure range used (0–1 bar), thus confirming that a mild increase in temperature is required to completely desorb CO_2_ at 25 °C. Under post combustion scenarios (higher temperatures), this increase is not necessary, recovering the maximum CO_2_ capacity by applying only pressure swing.

## 4. Conclusions

In this work, the characteristics and the behavior of mesoporous silica samples functionalized by grafting and by double functionalization were analyzed, in order to evaluate in energetic terms their performance as CO_2_ capture material in post combustion scenarios. 

The maximum value of thermokinetic parameter for the functionalized samples indicated that the dominant mechanism depends on the amine density. For the sample with low/medium amino groups density (4–5 molec·nm^−2^), the carbamic acid/silyl carbamate formation would be the mechanism that is dominant. For the double functionalized sample (MSG20I30, high amino density), CO_2_ diffusion would be the limiting phenomenon. 

The microcalorimetric studies confirmed that new adsorption sites were generated by the functionalization step. For materials with higher amine density, the proportion of propyl carbamate/silyl carbamate formed is higher than for materials with low or medium amine density. This is agreement with the distribution of sites found from the differential adsorption enthalpy of MSG20I30. This sample did not present a signal of silyl formation (stronger bonds that could cause irreversibility in cycles). 

CO_2_ adsorption capacities increased with the temperature for MSG20I30 sample, an opposite behavior than the MSG20 sample. This fact suggested a greater contribution of physisorption mechanism than CO_2_ chemisorption on MSG20. These properties derived in higher selectivity, higher working capacity, and also higher API values for MSG20I30 than MSG20 sample at high temperatures (50 and 75 °C).

A complete desorption of MSG20I30 at 25 °C was not possible only by molecular vacuum. The differential adsorption enthalpy at zero coverage suggests that this irreversibility is attributed to the occupation of sites that are not restored after the first adsorption round, changing the sites distribution on the sample. This occupation of sites could be caused by either diffusional limitation or strong chemical bonds of adsorption products formed. At higher temperatures, these sites become free after the first outgassing process. 

Thereby, from the obtained results, the double functionalization method would be a more efficient route to incorporate amino groups on the support with views to its application on post combustion scenarios under dry conditions, taking into account the less consumption of energy to recover the maximum CO_2_ capacity and its higher performance at high temperatures.

## Figures and Tables

**Table 1:** Elemental analysis of the samples studied.

Sample	C (%)	H (%)	N (%)	C (mmol·g^−1^)	N (mmol·g^−1^)	*C/N*	MTAC ^a^
MSS	0.24	0.42	0.04	0.21	0.03	-	-
MSG20	7.44	1.53	2.46	5.86	1.76	3.33	0.88
MSG20I30	21.08	5.39	10.62	17.57	7.59	2.31	3.80

^a^ Maximum theoretical adsorption capacity by chemisorption, mmol CO_2_ g^−1^.

**Table 2:** Textural properties calculated from N_2_ adsorption/desorption isotherms.

Samples	A_BET_ (m^2^·g^−1^)	Pore Vol (cm^3^·g^−1^)	Pore Size (nm)	Microp Vol (cm^3^·g^−1^)
MSS	392	1.43	9.6	0.125
MSG20	211	0.96	7.8	0.056
MSG20I30	52	0.06	7.7	0.014

A_BET_: specific surface area as determined by Brunauer-Emmett-Teller (BET) equation; Pore Vol: total pore volume, as calculated from adsorbed N_2_ at P/P_0_ ~ 0.985; Microp Vol: total micropore volume, as determined by D-R equation.

**Table 3:** Amine density related to calorimetry characterization results.

Samples	Ø_−NH2_^a^ molec·nm^−2^	Energy Sites μmol CO_2_g^−1^		Thermokinetic Parameter	
		<40 kJ mol^−1^	>40 kJ mol^−1^	τ_max_, s	Δ*H*_ads_ kJ mol^−1^
MSG20	5.02	190	573	354	-43
MSG20I30	87.91	240	1113	1274	-34

^a^ Assuming a homogenous coverage.

**Table 4:** Fitting parameters to the experimental data for MSG20 at 50 °C and 75 °C.

Parameter	CO_2_		N_2_	
	50 °C	75 °C	50 °C	75 °C
*q_m1_*, mmolg^−1^	0.76	0.68	0.57	0.43
*b_1_*, bar^−1^	9.41	9.42	0.13	0.08
*q_m2_*, mmolg^−1^	3.28	2.65		
*b_2_*, bar^−1^	0.05	0.04		
R^2^	0.9812	0.9991	0.9274	0.9421

**Table 5:** Fitting parameters to the experimental data for MSG20I30 at 50 °C and 75 °C.

Parameter	CO_2_		N_2_	
	50 °C	75 °C	50 °C	75 °C
*q_m1_*, mmolg^−1^	2.13	2.66	0.016	0.015
*b_1_*, bar^−1^	14.00	15.70	0.94	0.91
*q_m2_*, mmolg^−1^	1.02	1.06		
*b_2_*, bar^−1^	0.13	0.11		
R^2^	0.9739	0.9970	0.9795	0.9825

**Table 6:** Comparison of adsorption capacity of MSG60 and MSG20I30 with others similar ones found in the literature.

Sample	T (°C)/p_CO_2__ (bar)	Amine, N Content (mmol/g)	CO_2_ Uptake (mmol/g)	Reference
SBA-15	60/0.15	APTES, 1.89	1.06	[54]
SBA-15	60/0.15	APTES, 2.70	0.52	[55]
SBA-15	60/0.15	APTES, 2.61	0.66	[32]
CC *	45/0.25	MAP, 2.63	0.87	[56]
MCM-41	45/0.25	MAP, 3.42	1.10	[56]
MSG20	50, 75/0.15	APTES, 2.46	0.59/0.44	This work
SBA-15	45/1	APTES/PEI, 7.64	2.52	[22]
SBA-15	45/1	APTES/TEPA, 7.92	3.16	[57]
SBA-15	45/1	APTES/PEI, 7.00	1.88	[57]
PQCS2129 **	50,80/0.15	APTES/PEI,7.50	2.91/2.31	[58]
MSG20I30	50, 75/1	APTES/PEI, 7.59	2.1/2.61	This work

* Commercial Silica gel; ** Commercial Silica support.

**Table 7:** Working capacity, CO_2_/N_2_ selectivity, adsorption enthalpy, and Adsorbent Performance Indicador (API) values at 50 and 75 °C for MSG20I30 and MSG20 samples.

Sample	T (°C)	WC (cm^3^·cm^−3^) 0.02–1 bar	α, CO_2_/N_2_	Δ*H*_ads_, (kJ mol^−1^)	API
MSG20I30	50	15.6	1453.4	73.2	309.7
	75	17.4	1679.8		399.1
MSG20	50	11.83	194.47	43.8	52.2
	75	11.55	237.57		62.3
